# A Sensor for the Measurement of the Moisture of Undisturbed Soil Samples

**DOI:** 10.3390/s130201692

**Published:** 2013-01-29

**Authors:** Goran Kitić, Vesna Crnojević-Bengin

**Affiliations:** Faculty of Technical Sciences, University of Novi Sad, Trg Dositeja Obradovića 6, 21000 Novi Sad, Serbia; E-Mail: bengin@uns.ac.rs

**Keywords:** soil moisture sensor, sensor modeling, permittivity measurement

## Abstract

This paper presents a very accurate sensor for the measurement of the moisture of undisturbed soil samples. The sensor relies on accurate estimation of the permittivity which is performed independently of the soil type, and a subsequent calibration. The sensor is designed as an upgrade of the conventional soil sampling equipment used in agriculture—the Kopecky cylinder. The detailed description of the device is given, and the method for determining soil moisture is explained in detail. Soil moisture of unknown test samples was measured with an absolute error below 0.0057 g/g, which is only 2.24% of the full scale output, illustrating the high accuracy of the sensor.

## 1. Introduction

Exact measurement of soil moisture is needed in variety of applications. In agriculture, proper growth, development and maintenance of the plants depend very significantly on soil water content. Other applications range from monitoring of soil water content in various types of soil studies or in ecosystem management, to geo-engineering where high soil water content can indicate potential danger of landslides. They all have in common a great need for precise measurement of the soil water content.

Soil water content measurement can be performed *in situ*, or a sample can be measured in the laboratory using various techniques. However, both methods involve some disturbance to the measured soil, either by inserting the sensor into the ground or by handling the sample. Although some miniature *in situ* sensors exist which tend to minimize soil disturbance during insertion [1–8], their accuracy and reliability depend on the soil type and the use of the calibration process. Furthermore, characteristics of some sensors degrade with time [9,10]. All this significantly limits the applicability of *in situ* sensors in real-life scenarios. On the other hand, the most often used laboratory method for soil water content measurement is based on drying of the sample and measurement of the resulting mass decrease [11]. Although this method leads to very accurate results and requires standard laboratory equipment (oven and precise scale), it is very time consuming, as it lasts around 24 h. It also destroys the soil sample and allows no repeatability of the measurement. The purpose of this work was to develop a reliable and accurate method for laboratory measurement of soil water content of undisturbed samples of arbitrary soil type, that requires no drying and can be repeated as many times as needed.

In Section 2 we present the design of the proposed sensor device, based on the upgrade of the typical equipment for sampling of soil in an undisturbed state—the Kopecky cylinder. The operational principle of the proposed device is explained in detail in Section 3. The operational frequency range is determined as the one over which precise characterization of the electric properties of the soil is possible. The accurate transmission-line model of the device is presented and then simplified using lumped elements, to obtain an analytical relation between the measured reactance of the device and the permittivity of the sample. Section 4 presents a procedure for the extraction of precise values of the actual permittivity and effective conductivity of the sample from the measured results, which is independent of the soil type. The procedure is validated using various liquid and granular materials with known permittivity. In Section 5, the calibration curves of the sensor are developed for two types of soil, clay loam (humogley) and sandy clay loam (carbonate chernozem), which relate actual soil permittivity with their water content. The proposed sensor has been tested using four unknown soil samples of both soil types. The conclusions are presented in Section 6.

## 2. Sensor Design

Soil water content can be determined by measuring the permittivity of the sample [12]. Since relative permittivity of dry soil is about 2.5, and permittivity of water is 80, even a small amount of moisture causes significant changes in the permittivity of the soil. This dependence allows the design of soil moisture sensors based on permittivity measurement.

However, it should be noted that the soil permittivity strongly depends on its bulk density. Therefore, to accurately measure soil moisture, the disturbance to the soil as a result of sampling should be kept to the minimum. Typically, undisturbed soil sampling in agriculture is performed using a Kopecky cylinder, a stainless steel cylinder with diameter *D_c_* = 54 mm and height *H_c_* = 44 mm. The Kopecky cylinder is directly introduced into the soil by applying force to its upper rim. The bottom rim of the Kopecky cylinder is designed to minimize physical disturbance of the sample, ensuring that its density will not differ from that found in the ground. If the sample were to be removed from the cylinder, especially in case of relatively low water content, it could crumble or fall apart, resulting in decreased density and, consequently, in changed permittivity. Therefore, the first demand that had to be met in the design of the proposed sensor was that the samples should not be removed from the Kopecky cylinder and that the cylinder itself should become a part of the measurement device. In this way, the existing sampling equipment can be easily upgraded to allow electric characterization of the samples as well. However, since Kopecky cylinders were designed to satisfy only certain mechanical constraints, their integration into an electrical device needed for the measurement of permittivity was not a straightforward task.

**Figure 1.**
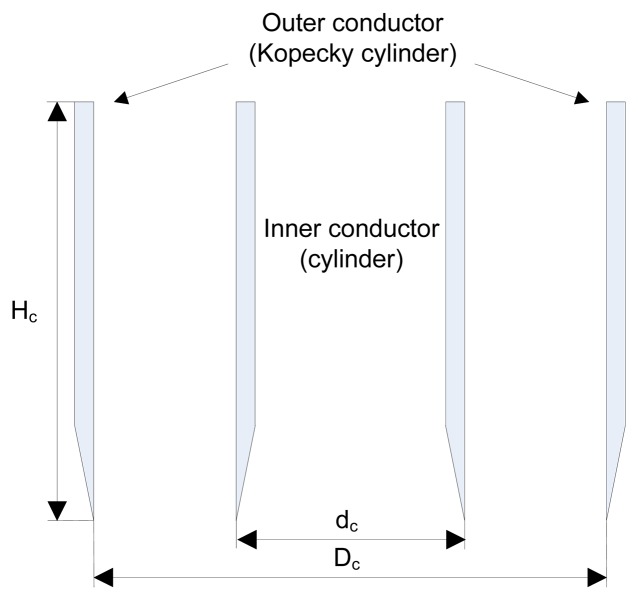
Cross-section of the Kopecky coaxial line – the central part of the proposed sensor.

In the proposed sensor, the Kopecky cylinder serves as an outer conductor of a coaxial transmission line realized by inserting a metal cylinder with a smaller diameter *d*_c_ into the middle of the soil sample. In this manner, a Kopecky coaxial line has been obtained. The cross-section of the proposed device is shown in Figure 1. The bottom rim of the inner cylinder has been designed to minimize the disturbance of the sample between two cylinders. The diameter of the inner cylinder *d*_c_ has been determined from the condition that the characteristic impedance of the Kopecky coaxial line, Equation (1), should be equal to 50 Ω:

(1) $$Z_{0}=\frac{1}{2\pi }\sqrt{\frac{\mu }{ɛ}}\ln(\frac{D_{c}}{d_{c}})=50\Omega$$

where *μ* and *ε* are the permeability and permittivity of the medium inside the Kopecky coaxial line, respectively. The 50 Ω condition is necessary to obtain impedance matching between the measurement device, standard 50 Ω SMA connector, and a Vector Network Analyzer (VNA), which is used to measure the input reactance of the sensor. The obtained diameter of the inner conductor is *d_c_* = 23.5 mm.

**Figure 2.**
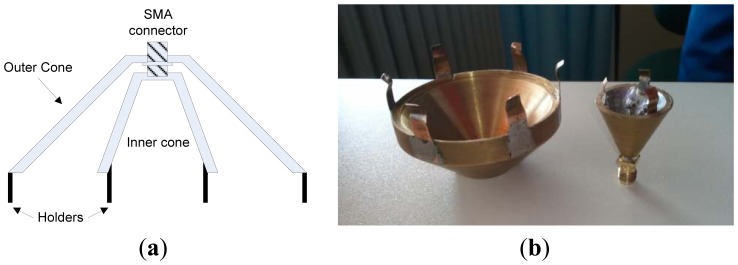
(**a**) Cross-section of the adapter (**b**) Photographs of two parts of the adapter.

To connect the Kopecky coaxial line to the SMA connector and the VNA probes, an adapter was designed, as shown in Figure 2. The adapter consists of two brass cones with a diameter ratio that fulfills the 50 Ω condition in Equation (1) at each cross section. At the top of the adapter a standard SMA connector is placed. Holders are mounted at the bottom end of both cones, to obtain a good electrical contact between the adapter and the Kopecky coaxial line.

**Figure 3.**
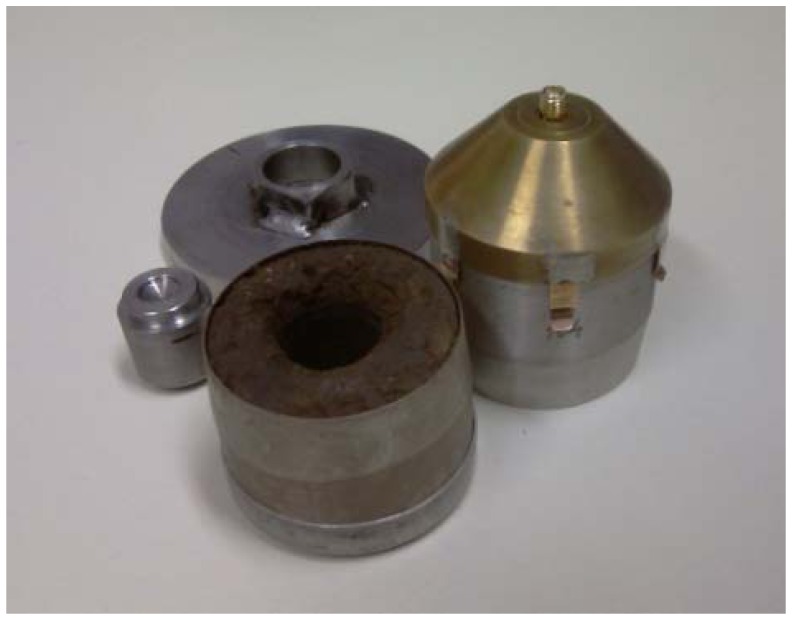
The proposed sensor (on the right) and its parts.

A photograph of the complete sensor and its parts is shown in Figure 3. Since the coaxial transmission lines are very sensitive to the mutual position of outer and inner conductor, a director (shown in the back) was used to align the axes of the inner and the outer cylinder exactly. In the front, the open Kopecky cylinder with its inner conductor removed is shown, while the completely assembled sensor is on the right hand side of the photo.

## 3. Operational Principle and Model of the Sensor

The capacitance of Kopecky coaxial line *C_C_* varies with soil permittivity and thus with soil moisture as well. However, this capacitance cannot be measured directly. Instead, the input reactance of the entire sensor *X_in_* can be measured by VNA. To obtain an analytical relation between the measured *X_in_* and the actual value of *C*_C_, an electrical model of the sensor has been developed.

We note here that measuring *X_in_* is not the only way to determine *C*_C_. For example, in [13,14] the complex reflection coefficient measured using VNA was used to determine the capacitance of the open-ended coaxial probe and, consequently, the permittivity of the sample. However, such an approach resulted in ambiguous solutions for the permittivity and a specific procedure had to be used to determine the valid one. The purpose of this work was to develop a fast, efficient and reliable method for non-ambiguous estimation of the permittivity. Since the water content predominantly influences the real part of permittivity, accurate determination of its imaginary part is not necessary. By relying on the measurement of input reactance of the sensor, we have developed a simple and straightforward method for estimation of the real part of permittivity, which yields unique solutions, thus avoiding the need for specific procedures for choosing between ambiguous solutions.

To allow the characterization of dielectric properties of the soil in a wider frequency range, we first analyze the operational frequency limits of the proposed sensor. The upper frequency limit is determined by the appearance of higher modes in the Kopecky coaxial line. The fundamental propagation mode is TEM mode, while the unwanted TE_11_ mode appears at the frequency:

(2) $$f_{c}=\frac{c_{0}}{\pi \frac{D_{c}+d_{c}}{2}\sqrt{\mu_{r}{ɛ}_{r}}}$$

where *c_0_* is the speed of light in vacuum. Above *f_c_*, two waves with different propagation constants would superimpose, resulting in deteriorated performances of the device [15]. For given dimensions of the sensor, and taking into account the theoretical maximal permittivity of humid samples (equal to 80), the upper frequency limit of the device is calculated to be 250 MHz. To exclude the response of capacitors at DC, the lower operating frequency has to be set above 0 Hz. Furthermore, the lower frequency limit is determined by the Maxwell-Wagner polarization which has a significant effect on the permittivity estimate of clay rich soils at low frequencies [16]. Therefore, the lower frequency limit of the device is set to 100 MHz.

**Figure 4.**
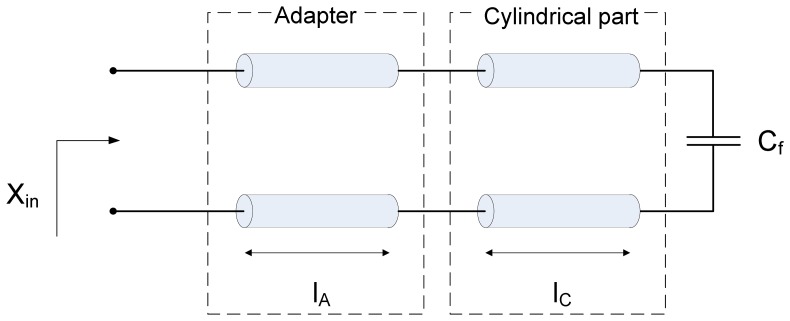
The transmission-line model of the proposed sensor. Lengths of the transmission lines are *l_c_* = *H_C_* = 44 mm, *l_A_* = 24.5 mm, fringing capacitance *C_f_* = 0.51 pF.

It should be noted that the upper limit of 250 MHz is set for measurement of soil permittivity only. The proposed device can be used to measure the permittivity of various liquid and granular materials. Depending on the expected value of the permittivity, the upper limit of the operating frequency range can be extended up to 1 GHz. In that case, operating frequencies are relatively high with respect to the dimensions of the sensor. Therefore, in order to accurately model the sensor, elements with distributed parameters *i.e.*, transmission lines should be used. The transmission-line model of the proposed sensor is shown in Figure 4.

The adapter and the Kopecky coaxial line are modeled by two serially connected transmission lines of certain lengths (Figure 4). Since the Kopecky coaxial line is open at one of its ends, the capacitor *C_f_* is included to account for fringing fields at the open end of the transmission line. The value of *C_f_* has been determined through numerical simulations in COMSOL Multiphysics 3.5 and it is equal to *C_f_* = 0.51 pF. As confirmed by additional simulations, *C_f_* is independent of the electric properties of the sample placed inside the Kopecky coaxial line.

**Figure 5.**
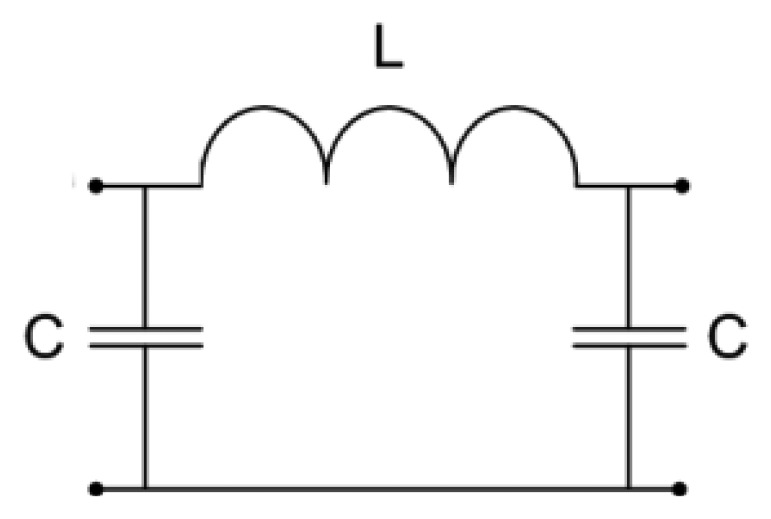
Lumped element model of an electrically short section of the lossless transmission line.

**Figure 6.**
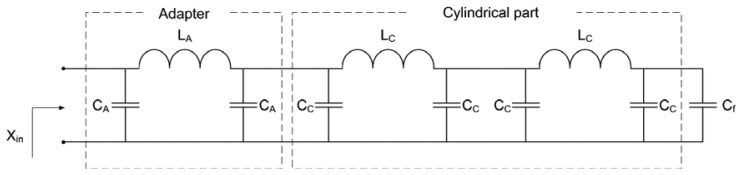
Lumped element model of the proposed sensor.

The initial model with distributed parameters can be simplified by replacing each transmission line with an appropriate lossless lumped element model. An electrically short section of the transmission line, approximately one quarter of the guided wavelength long, can be modeled using one LC section shown in Figure 5. Having in mind the physical length of the proposed sensor, two LC sections are needed to model the Kopecky coaxial line and one LC section to model the adapter. The final lumped element model of the proposed sensor is shown in Figure 6.

Values of the lumped elements in the model can be calculated using known expressions for inductance and capacitance of a transmission line [17]:

(3a) $$C=\sqrt{{ɛ}_{r}}\frac{\tan\left(\frac{\omega }{c_{0}}\frac{l}{2}\sqrt{{ɛ}_{r}}\right)}{\omega Z_{0}}$$

(3b) $$L=\frac{1}{\sqrt{{ɛ}_{r}}}\frac{Z_{0}\sin\left(\frac{\omega }{c_{0}}l\sqrt{{ɛ}_{r}}\right)}{\omega }$$

where *l* is the length of the transmission line, *Z_0_* is its characteristic impedance, and *ω* is angular frequency.

From the lumped element model, input reactance of the device can be easily found as:

(4) $$X_{in}=-\frac{A}{B}$$

where *A* and *B* are:

(4a) $$A=\left\lfloor 1-\omega^{2}(L_{A}(C_{A}+C_{f})+2L_{C}C_{f})\right\rfloor +C_{C}\left[-4\omega^{2}(L_{A}+L_{C})+2\omega^{4}L_{C}(2L_{A}(C_{A}+C_{f})+L_{C}C_{f})-2\omega^{6}L_{A}{L_{C}}^{2}C_{A}C_{f}\right]+{C_{C}}^{2}\left[2\omega^{4}L_{C}(3L_{A}+L_{C})-2\omega^{6}L_{A}{L_{C}}^{2}(C_{A}+C_{f})]-{C_{C}}^{3}[2\omega^{6}L_{A}{L_{C}}^{2}\right]$$

(4b) $$B=\left\lfloor \omega (2C_{A}+C_{f})-\omega^{3}C_{A}(L_{A}C_{A}+4L_{C}C_{f})+2\omega^{5}L_{A}L_{C}{C_{A}}^{2}C_{f}\right\rfloor +C_{C}\left[4\omega -\omega^{3}(L_{A}(4C_{A}+C_{f})+4L_{C}(2C_{A}+C_{f}))+4\omega^{5}L_{C}C_{A}(L_{A}(C_{A}+C_{f})+L_{C}C_{f})-2\omega^{7}L_{A}{L_{C}}^{2}C_{A}C_{f})\right]+{C_{C}}^{2}\left[-6\omega^{3}L_{C}+2\omega^{5}L_{C}(3L_{A}C_{A}+L_{A}(2C_{A}+C_{f}))-2\omega^{7}L_{A}{L_{C}}^{2}C_{A}(C_{A}+C_{f})\right]+{C_{C}}^{3}[2\omega^{5}{L_{C}}^{2}-2\omega^{7}L_{A}C_{A}{L_{C}}^{2}]$$

The input reactance is a function of angular frequency *ω* = *2πf*, as well as of all elements of the model: *C_A_*, *L_A_*, *C_C_*, *L_C_* and *C_f_*. However, only *C_C_* depends on the soil permittivity, since it can be assumed that the inductance of a short section of transmission line in Equation (3b) does not depend either on the permittivity of the medium or on the operating frequency:

(5) $$L\approx \frac{1}{\sqrt{{ɛ}_{r}}}\frac{Z_{0}\frac{\omega }{c_{0}}l\sqrt{{ɛ}_{r}}}{\omega }=\frac{Z_{0}l}{c_{0}}$$

Therefore, *L_A_* and *L_C_* can be regarded as constant over the whole frequency range of interest and for all measured materials.

The capacitance of the adapter *C_A_* is given by Equation (3a). Since the adapter is always air-filled, the capacitance *C_A_* does not depend on the permittivity of the sample. In the case of measurement of soil permittivity, where operating frequencies range from 100 MHz to 250 MHz, frequency dependency of *C_A_* can also be neglected since it influences *C_A_* only to the amount of 0.1%.

Using Equations (3a) and (3b), the following values of the lumped elements of the model have been obtained: *L_A_* = 4.075 nH, *C_A_* = 0.819 pF, and *L_C_* = 3.61 nH. When these values are substituted in Equations (4a) and (4b), the dependence between the input reactance and the sample capacitance *C_C_* is obtained:

(6) $$X_{in}=-\frac{A}{B}$$

where *A* and *B* are:

(6a) $$A=1-3.5917\cdot 10^{-7}f^{2}+C_{C}\left(-1.2136\cdot 10^{-6}f^{2}+0.1426\cdot 10^{-12}f^{4}-2.7296\cdot 10^{-21}f^{6}\right)+{C_{C}}^{2}\left(1.7819\cdot 10^{-13}f^{4}-8.6851\cdot 10^{-21}f^{6}\right)-{C_{C}}^{3}\cdot 6.5351\cdot 10^{-21}f^{6}$$

(6b) $$B=1.3496\cdot 10^{-5}f-2.1741\cdot 10^{-12}f^{3}+2.1741\cdot 10^{-12}f^{3}+9.856\cdot 10^{-20}f^{5}+C_{C}\left(2.5133\cdot 10^{-8}f-11.5207\cdot 10^{-12}f^{3}+8.4042\cdot 10^{-19}f^{5}-1.4047\cdot 10^{-26}f^{7}\right)+{C_{C}}^{2}\left(-5.3728\cdot 10^{-12}f^{3}+1.2561\cdot 10^{-18}f^{5}-0.0477\cdot 10^{-24}f^{7}\right)+{C_{C}}^{3}\left(2.5524\cdot 10^{-53}f^{5}-3.3629\cdot 10^{-26}f^{7}\right)$$

*f* is operating frequency in MHz, and *C_C_* is given in pF.

**Figure 7.**
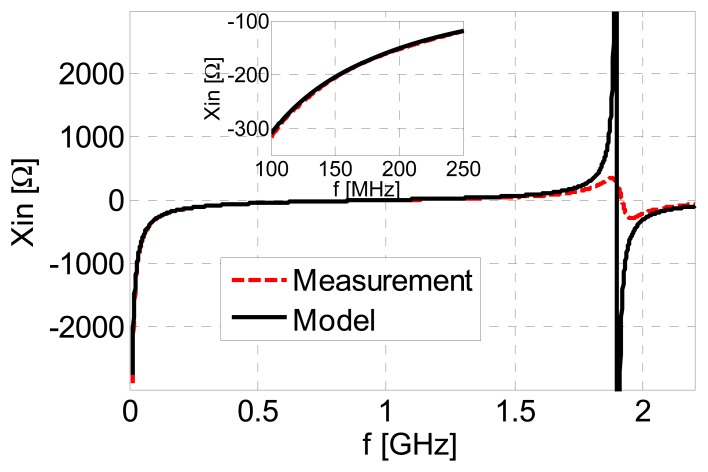
Comparison between the measured input reactance of the sensor, and the input reactance of the lumped-element model, in the case of the air-filled device.

To validate the model, Figure 7 shows a comparison between the measured input reactance and the input reactance of the model calculated using Equation (4), for the air-filled device. The comparison has been performed in a very wide frequency range, up to 2 GHz. A very good agreement can be observed, illustrating the validity of the model. The difference in the magnitudes of the responses around 1.9 GHz is solely due to the fact that the lumped model does not include any losses. However, this does not diminish its validity, even more so since the resonance around 1.9 GHz is out of the operational frequency range of the proposed sensor.

## 4. Permittivity Measurement and the Influence of Losses

The input reactance of the sensor filled with a material under test is measured over a range of frequencies using VNA. As mentioned above, the upper limit of this frequency range depends on the actual value of the permittivity, and it extends to 1 GHz for materials with permittivity close to 1. Using the measurement results and the expressions Equations (6) and (3a), the permittivity of the sample is then obtained as a function of frequency, independently of the type of the material under test.

**Figure 8.**
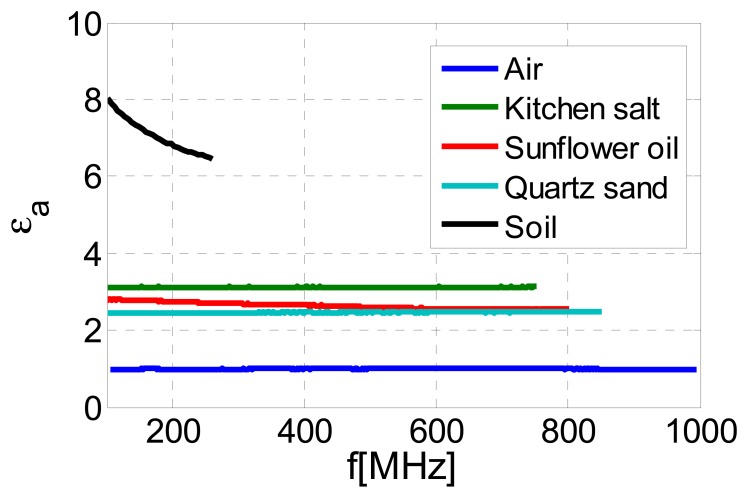
Measured permittivity of various samples, illustrating that the proposed device can be used to estimate permittivity independently of the type of material under test.

To illustrate this, the permittivity of various samples has been determined, Figure 8. It can be seen that the measured permittivity of air, kitchen salt, sunflower oil and quartz sand correspond very well to the actual values [18–20]. Furthermore, the measured permittivity of these materials is constant over the frequency range of interest. However, a frequency dependence of permittivity is observed in the case of sunflower oil (weak) and a humogley soil sample with unknown water content (much stronger), due to the fact that these samples exhibit dielectric losses which have not been taken into account by the model.

The permittivity obtained from the measured input reactance and shown in Figure 8 is in fact the apparent permittivity *ε_a_*, which is a function of frequency, of the actual relative permittivity *ε_r_* (which is constant over the frequency range of interest), and of the effective conductivity *σ_e_* (which accounts for the actual conductivity of the sample as well as for dielectric losses in the sample *i.e.*, for non-zero imaginary part of the permittivity) [12]. The actual permittivity *ε_r_* and the effective conductivity *σ_e_* can be extracted from the measured apparent permittivity by a fitting procedure, using [21]:

(7) $${ɛ}_{r}={ɛ}_{a}-\frac{\sigma_{e}}{4{ɛ}_{a}{{ɛ}_{0}}^{2}\omega^{2}}$$

Although initially proposed for time-domain measurements [21], we show that the expression Equation (7) can be successfully used in frequency domain as well, to model the influence of the losses on measurements. We note here that, theoretically, the proposed procedure could be performed without using Equation (7), by measuring complex input impedance of the sensor instead of its input reactance. However, in that case, the model presented in Figure 6 would have to include series resistors and shunt conductors, to account for the losses. This would in turn lead to final expressions far more complicated than Equation (6), from which it would not be possible to easily calculate the capacitance *C_C_*. Therefore, we have opted to use the lossless model, to easily calculate *C_C_* and *ε_a_*, and then to use Equation (7) to account for the influence of the losses.

By using the proposed approach, values of *ε_r_* and *σ_e_* have been obtained for all measured samples, and they are shown in Table 1. The obtained values correspond very well to the actual ones [18–20]. In the case of air, kitchen salt and quartz sand, the actual and apparent permittivity are practically identical, due to the fact that these materials are all very good dielectrics and exhibit a conductivity close to zero.

## 5. Calibration and Testing

In the previous Section, we have shown that the proposed sensor can be used to measure the actual relative permittivity *ε_r_* and the effective conductivity *σ_e_*, independently of the material under test. However, to use the proposed sensor to correctly estimate soil moisture, the measured permittivity *ε_a_* has to be correlated with the actual soil moisture values. A calibration procedure has to be performed which relies on the fact that the soil water content influences the mass of the sample as well as its dielectric properties. To that end, gravimetric water content *θ_g_* [22] has been used, defined as the ratio between mass of the water *m_w_* and the mass of the dry soil *m_ds_*:

(8) $$\theta_{g}=\frac{m_{w}}{m_{ds}}=\frac{m_{ws}-m_{ds}}{m_{ds}}$$

where the mass of the wet soil sample is denoted with *m_ws_*. The curves that relate the apparent permittivity and the gravimetric water content of the soil sample have been obtained using samples of humogley soil taken from the depth of 20 to 25 cm (humogley is a very fertile typical agricultural soil, which, according to USDA classification, belongs to clay loam type of soils. The samples were taken from the region of Banat in north-east Serbia.)

The calibration process started with a completely dry undisturbed soil sample placed in the Kopecky cylinder. The mass of the dry soil was first measured. The sample was subsequently immersed in distilled water for 48 h, to allow the saturation to be achieved by capillary effect. The saturated sample was weighed again. The inner conductor was inserted, and the input reactance was measured. The sample was left to dry and new measurements of input reactance were performed whenever the sample lost 2 g of its weight, until it became completely dry. In this way, a total of 19 measurements were performed. For each measurement, the apparent permittivity was extracted over the frequency range from 100 to 250 MHz.

**Figure 9.**
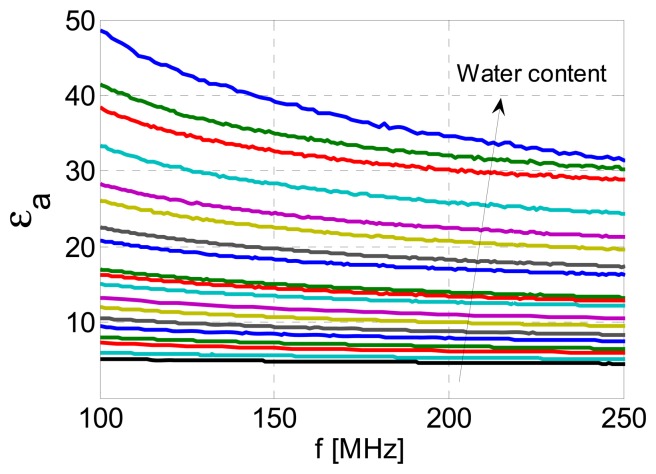
Apparent permittivity calculated from 19 measurements of the humogley soil sample with known water contents. The arrow indicates the increase of water content.

The obtained values of apparent permittivity over a specified frequency range are presented in Figure 9, for all 19 measurements. We note here that very similar results were obtained for measurements of 10 soil samples taken from the same location. As expected, higher water content resulted in higher average apparent permittivity over the frequency range of interest. Also, in all cases a frequency-dependence of apparent permittivity can be observed.

Using Equation (7), *ε_r_* and *σ_e_* can be extracted from the measured apparent permittivity. The actual permittivity *ε_r_* obtained in this way does not depend on the soil conductivity and therefore the proposed approach yields good results for the permittivity independently of the soil type. The obtained values of *ε_r_* and *σ_e_* are shown in Table 2 for all nineteen measurements and they correspond very well to the typical values found in [21,23,24]. As could be expected, both the actual permittivity *ε_r_* and the effective conductivity *σ_e_* increase with the soil water content.

**Figure 10.**
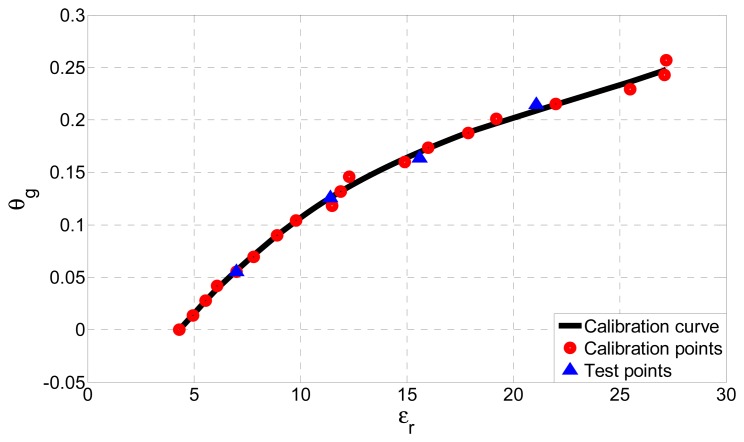
Calibration curve of the sensor (black line), which relates the actual permittivity of humogley soil to the gravimetric water content. The calibration curve has been created from 19 calibration points (red circles). Also shown are the results of 4 independent test measurements (blue triangles).

A calibration curve, shown in Figure 10, has been created by polynomial fitting of 19 calibration points from Table 2 (indicated with red circles in Figure 10), each determined by the value of the actual permittivity and the known water content.

The absolute error due to polynomial fitting is below 0.0094 g/g, *i.e.*, only 3.65% of the full scale output. To test the proposed soil moisture sensor, the input reactance of four unknown test soil samples with different gravimetric water contents was measured. The samples were taken from the same location and from the same depth (20–25 cm), as those used for calibration. Using the procedure described above and the calibration curve from Figure 10, gravimetric water contents *θ_g_* for all samples were determined. They were compared to the actual values of gravimetric water contents (also known), indicated in Figure 10 by blue triangles. The proposed soil moisture sensor proved to be very accurate, with the greatest absolute error equal to only 0.0057 g/g which is only 2.24% of the full scale output. Furthermore, the sensor produced accurate results for a wide range of soil moistures which include all values encountered in real-life scenarios (0–25%).

**Figure 11.**
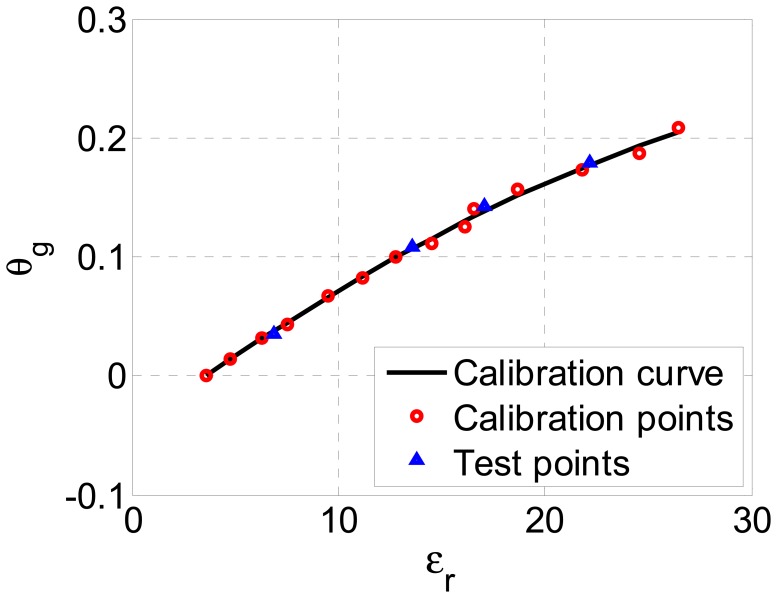
Calibration curve of the sensor (black line) for carbonate chernozem soil. Red circles denote calibration points, while blue triangles denote independent test measurements.

To investigate the applicability of the proposed sensor to various soil types, we have repeated the same measurement procedure for another set of soil samples. This time, samples of carbonate chernozem soil were measured, fertilized with 200 kg of nitrogen per ha, and taken from the depth of 10 cm. The obtained calibration curve is shown in Figure 11, with both calibration and test points denoted. Again, the proposed soil moisture sensor proved to be very accurate, with the greatest absolute error equal to only 0.0046 g/g, *i.e.*, only 2.22% of the full scale output.

## 6. Conclusions

A very accurate and robust sensor for measurement of soil moisture of undisturbed soil samples has been presented, based on an upgrade of the conventional soil sampling equipment used in agriculture—the Kopecky cylinder. Owing to the procedure developed, the sensor provides accurate permittivity measurements independently of the soil type. By using a calibration curve which relates the measured permittivity to the water content of the soil in question, the proposed sensor allows fast and repeatable estimate of soil moisture with an error below 2.24% with respect to the full scale output, over a wide range of soil moistures which include all values encountered in real-life scenarios (0–25%).

Our future work is aimed at the development of calibration curves for all typical types of soil in Serbia, including various types and quantities of applied fertilizers. Once finalized, such a bank of calibration curves will allow fast and straightforward measurement of soil moisture over a wide range of soil types, without the need to perform calibrations for each set of different samples.

It should finally be noted that the application of the proposed sensor is not restricted only to the measurements related to soil, *i.e.*, it can be used to accurately estimate the permittivity and effective conductivity of various other liquid or granular materials, independently of the type of the material used.

## Figures and Tables

**Table 1.** Actual relative permittivity *ε_r_* and effective conductivity *σ_e_* of various samples.

**Material**	***σ****_e_***[S/m]**	***ε****_r_*

Air	0	0.99
Kitchen salt	0	3.12
Sunflower oil	0.006	2.70
Quartz sand	0	2.46
Soil sample	0.081	10.7

**Table 2.** Actual relative permittivity *ε_r_* and effective conductivity *σ_e_* of the humogley soil, extracted from 19 measurements of the soil sample with different gravimetric water contents.

**No.**	***θ****_g_***[g/g]**	***σ****_e_***[S/m]**	***ε****_r_*

1	0.2569	0.359	27.2
2	0.2431	0.273	27.1
3	0.2292	0.246	25.5
4	0.2153	0.218	22
5	0.2014	0.182	19.2
6	0.1875	0.164	17.9
7	0.1736	0.136	16
8	0.1597	0.124	14.9
9	0.1458	0.100	12.3
10	0.1319	0.095	11.9
11	0.1181	0.081	11.5
12	0.1042	0.075	9.8
13	0.0903	0.068	8.9
14	0.0694	0.060	7.8
15	0.0556	0.053	7.0
16	0.0417	0.043	6.1
17	0.0278	0.038	5.55
18	0.0139	0.028	4.95
19	0	0.023	4.32
